# Novel Bioinformatics Method for Identification of Genome-Wide Non-Canonical Spliced Regions Using RNA-Seq Data

**DOI:** 10.1371/journal.pone.0100864

**Published:** 2014-07-03

**Authors:** Yongsheng Bai, Justin Hassler, Ahdad Ziyar, Philip Li, Zachary Wright, Rajasree Menon, Gilbert S. Omenn, James D. Cavalcoli, Randal J. Kaufman, Maureen A. Sartor

**Affiliations:** 1 Department of Computational Medicine and Bioinformatics, and Center for Computational Medicine and Bioinformatics, University of Michigan, Ann Arbor, Michigan, United States of America; 2 Sanford-Burnham Medical Research Institute, La Jolla, California, United States of America; 3 Department of Biostatistics, University of Michigan, Ann Arbor, Michigan, United States of America; 4 Morgridge Institute for Research, University of Wisconsin-Madison, Madison, Wisconsin, United States of America; 5 Departments of Internal Medicine and Human Genetics, and School of Public Health, University of Michigan, United States of America; 6 Institute for Systems Biology, Seattle, Washington, United States of America; King Abdullah University of Science and Technology, Saudi Arabia

## Abstract

**Setting:**

During endoplasmic reticulum (ER) stress, the endoribonuclease (RNase) *Ire1*α initiates removal of a 26 nt region from the mRNA encoding the transcription factor *Xbp1* via an unconventional mechanism (atypically within the cytosol). This causes an open reading frame-shift that leads to altered transcriptional regulation of numerous downstream genes in response to ER stress as part of the unfolded protein response (UPR). Strikingly, other examples of targeted, unconventional splicing of short mRNA regions have yet to be reported.

**Objective:**

Our goal was to develop an approach to identify non-canonical, possibly very short, splicing regions using RNA-Seq data and apply it to ER stress-induced *Ire1*α heterozygous and knockout mouse embryonic fibroblast (MEF) cell lines to identify additional *Ire1*α targets.

**Results:**

We developed a bioinformatics approach called the Read-Split-Walk (RSW) pipeline, and evaluated it using two *Ire1*α heterozygous and two *Ire1*α-null samples. The 26 nt non-canonical splice site in *Xbp1* was detected as the top hit by our RSW pipeline in heterozygous samples but not in the negative control *Ire1*α knockout samples. We compared the *Xbp1* results from our approach with results using the alignment program BWA, Bowtie2, STAR, Exonerate and the Unix “*grep*” command. We then applied our RSW pipeline to RNA-Seq data from the SKBR3 human breast cancer cell line. RSW reported a large number of non-canonical spliced regions for 108 genes in chromosome 17, which were identified by an independent study.

**Conclusions:**

We conclude that our RSW pipeline is a practical approach for identifying non-canonical splice junction sites on a genome-wide level. We demonstrate that our pipeline can detect novel splice sites in RNA-Seq data generated under similar conditions for multiple species, in our case mouse and human.

## Introduction

**Figure 1:**
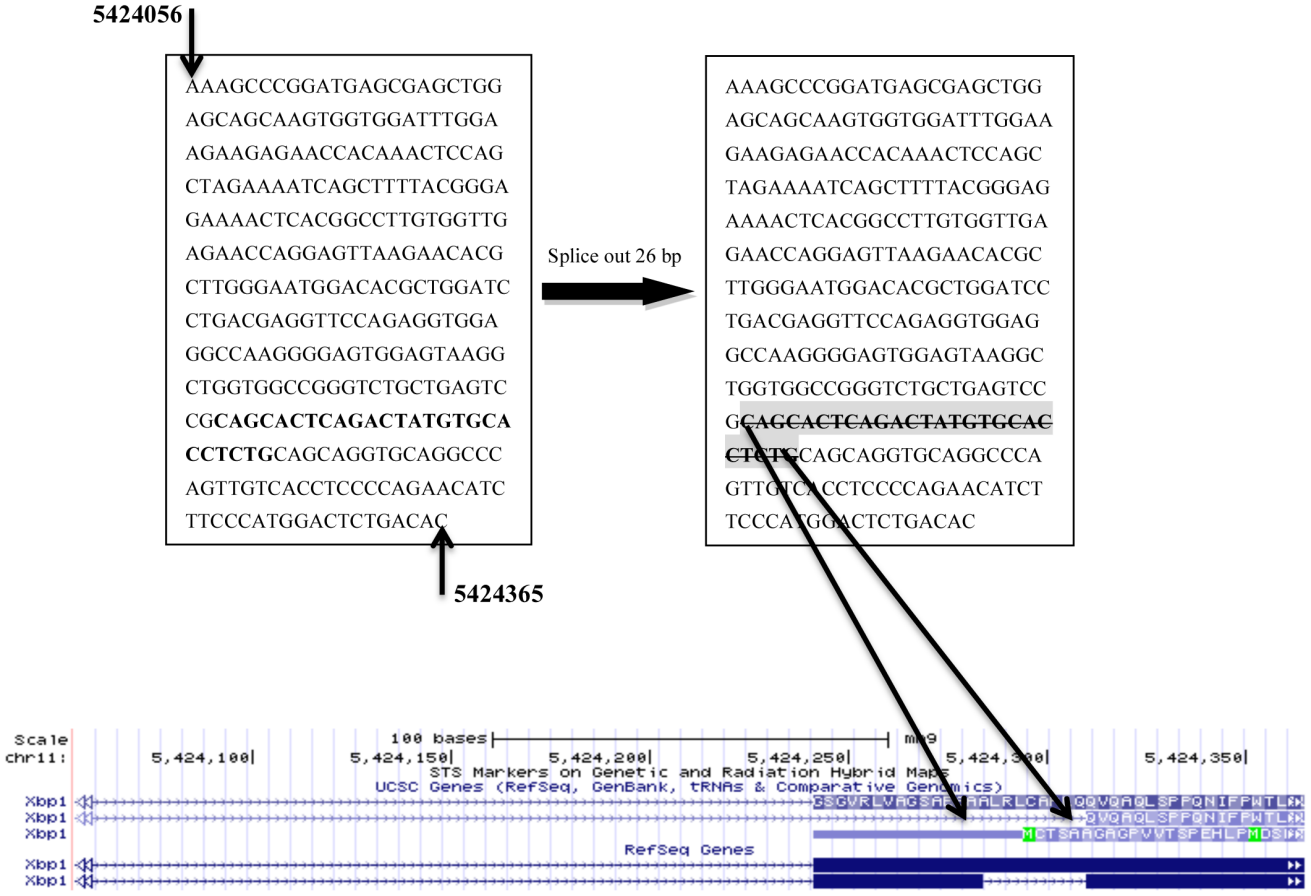
*Xbp1*'s partial transcript sequence, including the non-canonical 26 nt spliced region (highlighted on right).

The endoplasmic reticulum (ER) is the cellular organelle responsible for protein folding and assembly of approximately one-third of all eukaryotic cellular proteins [1]. The Unfolded Protein Response (UPR) is an adaptive intracellular stress response activated by the accumulation of unfolded or misfolded proteins within the ER lumen [2] and several reports indicate that ER stress is associated with diseases such as type II diabetes (T2D), metabolic disease, obesity, inflammation, neurodegeneration and cancer [3]–[6]. Upon activation of the UPR, the protein *Ire1*α (*Inositol Requiring Enzyme 1* α); also known as *ERN1* (endoplasmic reticulum to nucleus signaling 1), initiates the cytosolic (non-canonical) splicing of *Xbp1 (X-box binding protein 1)* mRNA to remove a 26 nucleotide (nt) sequence that results in a shift of the translational-open reading frame [7]. Spliced *Xbp1* mRNA encodes a potent transcriptional activator of hundreds of UPR genes encoding functions that facilitate protein folding, secretion, and degradation in response to ER stress. Figure 1 shows the 26 nt sequence (highlighted in gray) – chr11:5424282–5424307 (reference genome mm9) for the National Center for Biotechnology Information (NCBI) RNA reference sequence (RefSeq) No. NM_013842.3 spliced from the *Xbp1* gene.

Although the splicing of introns is inherently stochastic in terms of the mRNA sequence(s) required to define the 3′ and 5′ intron boundaries, there does exist a splicing code that predicts these boundary locations. Most splicing events fall into one of two highly conserved splicing categories: U2- and U12-dependent. Because U2 represents ∼99% of human introns, U12 intron splicing is commonly referred to as non-canonical splicing [8]. However, U2- and U12- dependent splicing events occur within nuclear pre-mRNAs whereas IRE1α-dependent *Xbp1* mRNA splicing occurs within the cytosol and has its own sequence recognition requirements [9]–[10], thus it is not only non-canonical in the sense of not being U2-dependent; it is uncharacteristic of any classification.

In recent years, new sequencing technologies for expressed RNA (RNA-Seq [11]) have improved expression profiling. Advantages of RNA-Seq compared to microarrays include the ability to detect the entire transcriptome and increased sensitivity for detecting splice variants, splice junctions, and novel transcripts. RNA-Seq can be performed on any of several technology platforms; our report focuses on data from the Illumina Genome Analyzer, however, our approach is applicable to other platforms. After the expressed reads are sequenced, they can be aligned against the reference genome plus junctions between known exon boundaries to include reads that cross splice junction sites. However, this approach excludes reads that cross non-canonical or unknown splice sites, which are interpreted as having “gaps” in them. We hypothesized that this set of unmappable reads could thus contain non-canonical and novel sequences, such as the region surrounding the 26 nt intron in *Xbp1*. Since the intron spliced from *Xbp1* mRNA is a non-canonical splice event, it serves as an ideal positive control around which to design our RSW algorithm. *Ire1*α null mouse embryonic fibroblast (MEF) cells provided a robust negative control, as they cannot produce the non-canonical splice in *Xbp1* mRNA.

Several methods exist to identify novel splice sites in RNA-Seq data, including Alt Event Finder [12], TrueSight [13], SplicingCompass [14], and PASTA [15]. Alt Event Finder [12] processes the mapped reads to report splice regions yet does not consider unmapped reads in the analysis input. Although TrueSight [13] utilizes all identified junctions and builds a regression model to report the best alignment, the algorithm is not suitable for our *Xbp1*-like non-canonical spliced region detection study due to the lack of pre-known *Xbp1*-like spliced regions for model training. SplicingCompass [14] maps reads to the reference genome initially and also does not utilize unmapped reads. It then predicts genes that undergo differential splicing based on the expression level, which is not applicable for identifying *Xbp1*-like spliced regions. PASTA [15] uses a combination of heuristic and statistical methods to identify canonical exon-intron junctions with high accuracy, especially at low coverage levels. PASTA [15] also uses the unaligned reads to infer the exact location of junction boundaries. Tophat also focuses on canonical exon-exon splice sites as well. In summary, none of these tools were designed for detecting the type of non-canonical, short splicing pattern observed in IRE1α-dependent *Xbp1* mRNA splicing. Many of these tools (TrueSight [13], SplicingCompass [14], PASTA [15]) also make use of paired-end sequencing data. However, in the case of *Xbp1* and similar cases, paired-end sequencing data would not be beneficial since the 26 nt length of the spliced region is within the error limits of the estimated insert length (i.e., a difference of 26 bp would be too small to reliably detect as significant). Thus, our method makes use of single-end sequencing reads.

With this motivation in mind, we developed an algorithm and pipeline called the Read-Split-Walk (RSW), to specifically process unmapped reads from RNA-Seq data to identify non-canonical, possibly very short, spliced regions; we then applied this pipeline to identify genome-wide *Ire1*α-dependent alternative spliced regions. The successful identification of the 26 nt *Xbp1* non-canonical splice site as the top hit in two separate experiments validates our algorithm and suggests no other mRNA splicing event dependent on IRE1α is as strong as *Xbp1*'s site under the contexts we examined. Although we only examined one time point, it was during the peak of IRE1α-dependent *Xbp1* mRNA splicing; thus, the lack of additional non-*Xbp1* mRNA splice targets is consistent with what was reported in yeast, where HAC1 was found to be the sole splicing substrate of IRE1α [16]. We compared our pipeline's results with related tools that might be used to detect novel non-canonical, short spliced regions such as BWA [17], Bowtie2 [18], and STAR [19]. In addition, we used known pattern detection (*grep*), and homology search approaches (Exonerate [20]) to estimate the sensitivity and precision of our RSW pipeline.

## Materials and Methods

### Origin of cell lines

Two mouse embryonic fibroblast (MEF) cell lines were developed for testing for IRE1α-dependent alternative spliced regions. In the first experiment using 35 bp reads, MEFs were prepared from day 9 embryos of Black 6 (B6) mice of greater than 10 generations back-crossed and of cell passage 6. IRE1α-dependent *Xbp1* mRNA splicing was knocked out using a floxed allele that removes the kinase domain and subsequently the RNase activity [21]. For the second experiment using 79 bp reads, MEFs were prepared from mice of the same B6 background except with a different null *Ire1α* knockout allele that results in a more complete gene deletion that removes both the kinase and RNase domains [22].

The human breast cancer cell line, SKBR3 was obtained from the American Type Culture Collection (Manassas, VA). The cells were maintained in culture with DMEM/F-12 medium supplemented with 10% FBS (Tissue Culture Biologicals, Seal Beach, CA) and 1% of Antibiotic-Antimycotic 100X (Gibco, Carlsbad, CA) [23]. The SKBR3 cell line is a model for the aggressive Her2/neu-amplified subtype of human breast cancers.

### Experimental design to detect *Ire1α* splicing targets

Mouse embryonic fibroblasts (MEFs) heterozygous (Het) *Ire1*α^+/−^ versus knockout (KO) *Ire1*α^−/−^ were treated for 4 hours with either 500 nM Thapsigargin (Tg) or 1 mM Dithiothreitol (Dtt) to induce the unfolded protein response (UPR). During these stresses, the Het cells are able to perform the non-canonical (26 nt) splicing of *Xbp1* mRNA, whereas *Ire1*α^−/−^ cells do not. Poly-A mRNA was isolated from these cells, sheared to approximately 250 nucleotides, randomly primed to form cDNA, and the Illumina linkers were added to the cDNA fragment library. Illumina sequencing (GAII) was performed on each sample. The Tg reads were 35 nucleotide single-end reads; the Dtt reads were 79 nucleotide single-end reads. The experiments with the 2 different treatments (Tg and Dtt) were conducted several months apart and changes in the Illumina platform resulted in differential read lengths and numbers of reads, allowing us to test RSW and compare the robustness of results under two independent, different sequencing approaches. These data have been deposited in NCBI's Gene Expression Omnibus (GEO) and are accessible through GEO Series accession number GSE54631.

### Initial read processing and reference genome selection

We used the Illumina (www.illumina.com) Genome Analyzer Pipeline Software package (GAII) to perform a complete data analysis of the four RNA-Seq datasets. Specifically, we employed the GAPipeline and the Off-Line Base caller (OLB) pipeline v1.6.1 suites of modules to perform image analysis (*Firecrest* module) and base calling (*Bustard* module) for the Genome Analyzer. We then collected the original read sequence files in *fastq* format and downloaded the mouse genome reference sequences (mm9) from the University of California Santa Cruz (UCSC) genome browser (http://genome.ucsc.edu). We also downloaded UCSC genes (knownGene.txt) from UCSC genome browser. The splice junction file was created by setting the sequence entry on each side of the junction site to 4 bp shorter than the read length (35 or 79 bp) using a RNA-Seq software python script (getsplicefa.py) from ERANGE version 3.1 (http://woldlab.caltech.edu/~alim/RNA-seq/). The original reference genome and splice junction site file were merged together to form an expanded genome.

### Identification of novel spliced regions using the RSW algorithm

#### Align read sequences to the mouse reference genome and split unmapped reads into halves

We used the Bowtie [24] aligner (version 0.12.7) to align original sequences against the expanded genome (mouse reference genome mm9 and known splice junction boundaries). We specified a maximum of 2 mismatches (bowtie parameter -v 2) and instructed Bowtie to report up to 11 valid alignments (-k 11). The aligner wrote reads with more than 10 reportable alignments (mapping locations) into a separate file (-m 10, —max). Since the goal of this project was to identify special non-canonical spliced regions, the “best” alignment option (—best) was also used. Likewise, we only processed original reads deposited in the unmapped read data set (—un option). The unmapped reads are used in the remainder of this report.

**Figure 2:**
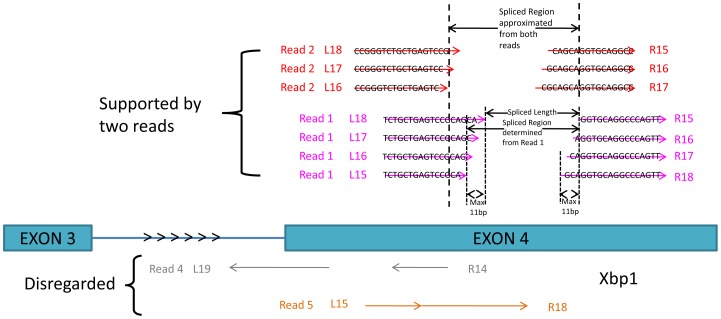
Illustration of a hypothetical potentially novel spliced region with two supporting reads. Below the gene are two examples that RSW would disregard: a split read pair mapped to the incorrect strand (gray) and a split read pair mapped with a 0 or 1 bp gap (orange).

Based on the overall average quality scores across the read length, we trimmed the last 2 bases of each read. Each unmapped read was used to create a small library of split pairs. The split pairs were generated by splitting the read at consecutive nucleotide positions, such that each end (left or right) of the read had a minimum of 11 and a maximum of 22 nucleotides if the total read length was 33 nucleotides (Tg treated samples) or a minimum of 11 and a maximum of 66 nucleotides if the total read length was 77 nucleotides (Dtt treated samples). Thus the sequential divisions between the left and right end across the length of the read produces a library of sequence pairs all coming from a single previously unmapped read; the resulting halves then undergo a new round of mapping that also keeps track of the gap/intron between halves (Figure 2). The RSW algorithm was performed on all unmapped reads from all samples.

#### Select split read halves mapped to genome and selection criteria for novel splice locations

We define a *split read pair* to be two sequences that were originally from the same read. Each split read half was then aligned back to the original reference genome and exon boundaries using Bowtie [24] under the same parameter settings except that no mismatch (option –v 0) was allowed for this alignment step. Following alignment, split read pairs were selected for follow-up analysis if they met certain criteria. We required that two split read ends map to within 40 kb of each other and map to the same strand and same chromosome, together with the same matched gene. We did not consider reads that were inversely mapped (right end mapped before left end on the + strand, or left before right on the - strand). We also filtered out pairs that were adjacent (spliced region length <2 bp), since this could occur based on sequence homology on either side of the split, and thus were likely false positives. The *spliced region* was defined as the interval between the nearest ends of the two split halves from the same read. The *splice length* was calculated as the distance between the alignment end position of the read split end having lower mapped genomic coordinates and the alignment start location of the read split end having higher mapped genomic coordinates.

All the selected split read ends for each read were consolidated into a unique *spliced region* if the genomic boundaries for their splice length were within a certain threshold (11 bp for Tg treated samples and 27 bp for Dtt treated samples) and their splice length was the same. These equivalently mapped reads were then consolidated into unique spliced regions. The final candidate spliced region for a gene was calculated as the range between the smallest lower boundary and the largest upper boundary among the regions of supportive reads where the splice length for every mapped read was the same (see Figure 2).

If a spliced region was within 5 bp of a known junction in the UCSC knownGene reference database or it was not supported by at least 2 independent reads, then it was not considered a novel candidate. Otherwise, the region was reported as a novel, candidate non-canonical region. We note that we report candidates supported by as low as 2 independent reads for completeness, so that users can observe the distribution of candidates by number of supported reads. These distributions in our data suggest that those supported by only 2–4 reads are likely false positives. Figure 2 illustrates the criteria we used for our algorithm for Tg treated samples.

To compare splice locations between the Het and KO cells, we allowed a 5 bp variance at either end of the splice. Clusters with multiple supporting reads in the Het sample but having no reads or very short splice length (<2 bp) in the KO sample were considered candidates. The results from both runs were combined and manually inspected for regions present across both treatments (Tg and Dtt). The step of selecting candidate split read pairs was performed in Oracle using SQL and the clustering algorithm was implemented in Perl. Scripts are available at http://sartorlab.ccmb.med.umich.edu/rsw.

#### Assessing the robustness of our choice of parameters

To test the robustness of our approach, we also assessed the effect of (1) changing the minimum split length (from 11 to 13), (2) changing the alignment parameter for mapping the split reads to allow 1 mismatch (original analysis allowed 0 mismatches), and (3) changing the minimum number of reads supporting a spliced region (from 2 to 5) for the Tg experiment. We did not assess the effect of changing the requirements that reads to map to the same strand, the same chromosome, and the same matched gene since these are critical requirements for our hypothesis.

#### Computational requirements for RSW

Computational requirements include Perl (v5.7 or later) and pre-installment of the alignment software bowtie (v0.12.7 or later) [24] in a location accessible in the path. In addition, the desired reference genome and relevant bowtie index files should be built. The UCSC refFlat.txt annotation file for the desired species should be in the same directory. At least 1 GB of RAM is recommended.

### BWA aligner to identify novel splices or gaps

We compared our algorithm to BWA [17] software, which uses gapped alignment, to test whether the known *Xbp1* 26 nt spliced region could be identified using BWA. We modified the BWA aligning parameters to allow for larger gaps, adjusting for the number of gap extensions (length of gap (-e from 26 to100 (maximum number of gap extensions), and altering the gap open penalty (-O 15–20) and the gap extension penalty (-E 0–4). Reported results are based on the BWA backtrack algorithm (Version 0.6.2-r126) “aln” command with -I (the input was in the Illumina 1.3+ FASTQ-like format), -O (gap open penalty was set to 15), -E (gap extension penalty was set to 0), and -e (the maximum number of gap extensions was set to 100). The BWA algorithm with the “samse” module was used to generate alignments. Results generated using the other above-mentioned parameters were the same.

### Bowtie2 aligner to identify novel splices or gaps

We additionally used bowtie2 [18], which as opposed to the original Bowtie allows gapped alignment of short reads. We used different combinations of gap open penalty (5, 7, 10, 15, 20, and 30) and gap extension penalty (0, 1, and 3) on Tg and Dtt data sets and compared the results with those from RSW.

### Running Exonerate and Unix “*grep*” command

In order to validate our RSW algorithm, we used the *Xbp1* 26 nt spliced region as a positive control case. To determine the total number of sequencing reads present in the heterozygous samples that spanned the 26 nt spliced region, we used a 33 bp DNA fragment flanking the novel 26 nt splice and identified reads aligning to this region using homology mapping (Exonerate [20]) and pattern matching (*grep*).

We ran Exonerate [20] version 2.2.0 in default mode (with perfect matches) as well as exploring different percent matched identification to account for 1–2 possible sequencing errors in the reads. Specifically, the command used was: Exonerate –percent 95 –showalignment FALSE –showvulgar false –ryo ‘%S\n’ FASTA1.fa FASTA2.fa. In the case of the search for *Xbp1* spliced regions, we used the normal *Xbp1* region or the spliced *Xbp1* for FASTA1.fa and the FASTA2.fa files, respectively, which contained the reads in “fasta” format. By default, Exonerate uses a local alignment model allowing for small gaps. The 26 gap is too large for Exonerate to identify in short sequencing reads; thus we ran it twice, once with the unspliced and once with the spliced version of *Xbp1*.

The Unix regular expression command *grep* was used to identify the exact pattern of the same 33 bp fragment spanning the 26 nt *Xbp1* splice in all of the unmapped reads by searching for the *Xbp1* 26 nt flanking sequence (concatenating upstream and downstream flanking sequences) with different combinations of read split pair lengths. The sum of these split read pair lengths is equal to the trimmed read length (33 for Tg treated samples and 77 for Dtt treated samples).

### Identification of novel spliced regions for SKBR3 using the RSW algorithm

In order to demonstrate that the RSW pipeline can be applied to sequencing data in other biological contexts and from humans, we employed RSW on RNA-Seq data from the human breast cancer cell line SKBR3 [23]. This study was conducted by the Chromosome 17 team of the Chromosome-centric Human Proteome Project (C-HPP) of the Human Proteome Organization (HUPO) [25]–[27]. HPP analyses involve integration of proteomics data into a genomic framework that will promote a better understanding of the relationship of the transcriptome to the proteome and of the pathways and biological networks involved in the phenotype [28]. Despite its relatively small size, chromosome 17 is rich in protein-coding genes, ranking second in gene density; it contains many cancer-associated genes, including BRCA1, ERBB2 (Her2/neu), TP53, and genes of the ERBB2 amplicon, making it an ideal region to use to test RSW.

Previously, splice variants expressed in the SKBR3 cell line were examined by integrating RNA-Seq and proteomic mass spectrometry data. From total RNA of SKBR3 cells, strand-specific RNA-Seq libraries were prepared according to Illumina TruSeq standard procedures. Each library was sequenced (101 bases, paired end) on 1–3 HiSeq 2000 lanes to obtain an average of 123 million uniquely mapped reads^4^. The mass spectrometric analyses on SKBR3 cell lysate samples were performed with an online Dionex nano-LC instrument coupled to a Fourier transfer mass spectrometer^4^. The criteria for splice variant identification used were the presence of unique reads aligning only to the transcript and matching peptide.

We used the same aligner, Bowtie [24], to align SKBR3 RNA-Seq reads against the expanded genome (human reference genome hg19 plus known splice junction boundaries) and used the same Bowtie parameter sets to obtain unmapped read sequences. We also trimmed the end two bases of each read. Again, we split unmapped read into halves, creating a small library of split pair reads. The split pairs were generated using a minimum of 33 and a maximum 66 nucleotides given that the total read length for the SKBR3 cell line RNA-Seq data was 99 nucleotides after trimming.

As performed for the mouse *Ire1*α study, we applied the same strategy of mapping read pairs to the genome and performed a similar SQL query to select mapped read pairs meeting certain criteria. Again, we removed false positive pairs (reads that were inversely mapped) and filtered out pairs that were adjacent. The spliced region and splice length were also defined similarly as our mouse analysis. For example, the same criteria (within 5 bp of a known junction in the UCSC knownGene reference database) was applied. The downstream read clustering and consolidation steps were implemented using the same algorithms/scripts. We performed separate analyses for the two ends of the paired-end RNA-Seq data and also ran RSW on the data set combining both ends of the paired-end data.

To check for the prevalence of splice regions detected by RSW in the SKBR3 cell line, we used the Ensemble annotation file (Homo_sapiens.GRCh37.74.chr17.gtf downloaded from http://useast.ensembl.org/info/data/ftp/index.html) to annotate the splice junction boundaries of 217 protein isoforms from 108 genes on chromosome 17 and then checked exon boundaries against candidate splice junction coordinates reported by RSW. To be called a match, the two identified splice junction boundaries were required to be within 5 bp from each other. The presence of at least one matched junction was required to claim an overlapped region between the two methods (proteomics and RSW).

## Results

### Mapping statistics

The Bowtie mapping statistics for Het samples in both experiments (Tg and Dtt) are shown in Table 1. The Dtt-treated Het sample contained both more total reads, and more unmapped reads when compared to the Tg-treated Het sample. The mapping statistics for KO samples were similar (not shown).

**Table 1:** Bowtie Mapping statistics for *Ire1*α^+/−^ samples treated with Tg and Dtt.

Sample Type	Read Length	Total # of reads	Total # of unmapped reads
Tg treated	35	8,922,329	418,002 (4.7%)
Dtt treated	79	23,442,681	2,055,557 (8.8%)

### Change in coverage at XBP1's non-canonical intron

**Figure 3:**
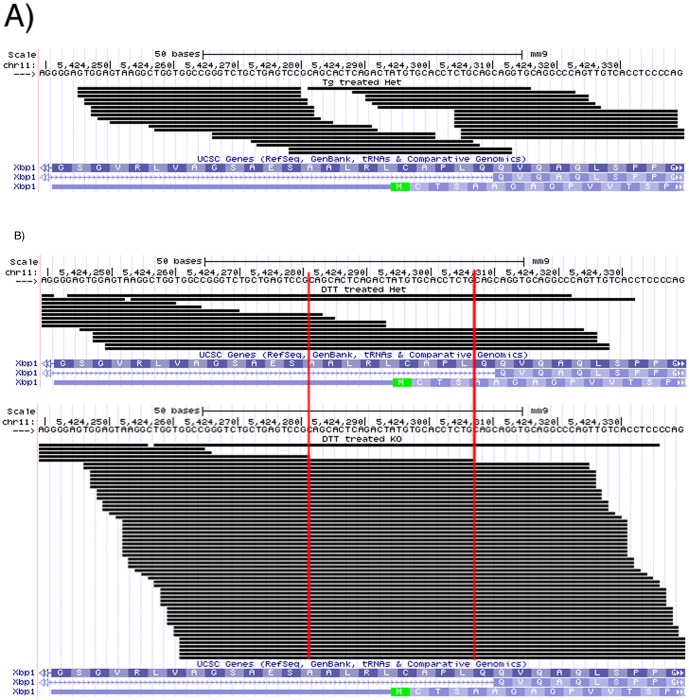
UCSC custom track view for *Xbp1* 26 nt spliced region in mouse embryonic fibroblast cells and qRT-PCR results. “Het”  =  *Ire1*α^+/−^; “KO”  =  *Ire1*α^−/−^. **a** = 500 nM Thapsigargin 4 hrs; **b** = 1 mM Dithiothreitol 4 hrs; For both a and b, Het reads are on top and KO reads are on the bottom. Red vertical lines indicate the 26 nt spliced region.

The aligned reads within and spanning the *Xbp1* 26 nt spliced region were visualized in a UCSC custom track (http://genome.ucsc.edu/) for both genotypes, and showed a distinct lack of reads mapped onto the 26 nt spliced region in the Het samples for both experiments (Figure 3). In contrast, this region was well covered throughout by reads in the KO samples.

### Novel spliced region results for XBP1 detected by RSW

The number of reads from the unmapped set that were identified as crossing over the *Xbp1* 26 nt region by the RSW algorithm were 21 and 173 for Tg and Dtt Het samples, respectively (Table 2). Actual lists of read halves and associated information supporting *Xbp1*'s 26 nt spliced region is reported in Table S1. This 26 nt spliced region in *Xbp1* was the top candidate region predicted by RSW for both experiments using the criteria described in the methods. For comparison, the next candidate for Tg was *Fhl1*, supported by only 4 reads with splice length of 1797 bp (not identified in Dtt), and the next candidate for Dtt was *Rps9*, supported by 80 reads with a splice length of only 4 bp (not identified in Tg). Using primers flanking the *Rps9* splice site on genomic DNA we confirmed the 4 nt gap is genomic in nature rather than being spliced at the mRNA level by IRE1α (not shown). Since no other candidate region met the criteria in both experiments, no other candidate genes were identified showing clear non-canonical spliced regions by *Ire1*α. The list of top ranked genes identified by RSW from the Tg and Dtt samples and their supporting read information is provided in Table S2.

**Table 2:** The number of reads aligned to *Xbp1*'s 26 bp splice junction by our “Read-Split-Walk” (RSW) algorithm for *Ire1*α^+/−^ and *Ire1*α^−/−^ samples.

Sample type	Gene	Splice length	Splice region	Supported by # of reads	
				Het	KO
Tg treated	*Xbp1*	26	chr11:5424280–5424312	21	0
DTT treated	*Xbp1*	26	chr11:5424280–5424312	173	0

### RSW results using alternative parameters

We assessed the effect of (1) changing the minimum split length from 11 to 13 bp, (2) changing the alignment parameter for mapping the split reads to allow 1 mismatch (original analysis allowed 0 mismatches), and (3) changing the minimum number of reads supporting a spliced region from 2 to 5. Increasing the minimum split read length to 13 still resulted in the *Xbp1* 26 bp splice site as the top ranked site, and supported by the same number of reads (21 for Tg and 173 for DTT treated samples). This is because the minimum split length that mapped to the borders of the *Xbp1* intron and no more than 9 other places was 14 bp (See Table S1). Allowing 1 mismatch (–v 1 parameter) in each split read resulted in zero read pairs selected to support the 26 bp region. This was due to the fact that many mates for read split pairs were filtered into the Bowtie max file having been mapped to >10 genomic locations when one mismatch was allowed. Requiring at least 5 supporting reads for a spliced region reduced the number of reported candidates from 55 to 1 for Tg (and from 2533 to 89 for Dtt), with no change in ranking since ranks were based on this criteria. While RSW by default reports candidates having as low as 2 supporting reads for completeness, those candidates supported by few reads should be viewed with scepticism. Inclusion of those supported by few reads in the report facilitates judging the significance of top hits such as *Xbp1*. These results suggest that the default running parameter settings for RSW work well.

### RSW results under the null expectation of no novel spliced regions

To assess the specificity of RWS, we ran our pipeline on a subset of the Tg data set for reads that mapped uniquely to the reference genome (chr19); in this case we do not expect to identify any novel spliced regions since the complete reads map to the genome. Indeed, we did not observe any novel spliced regions from this read set that met all of our criteria. (Several spliced regions known by UCSC were identified and filtered.) Extending this to the full genome suggests we can expect no more than a minimal number of false positives.

### RSW sensitivity compared with alternative approaches

**Figure 4:**
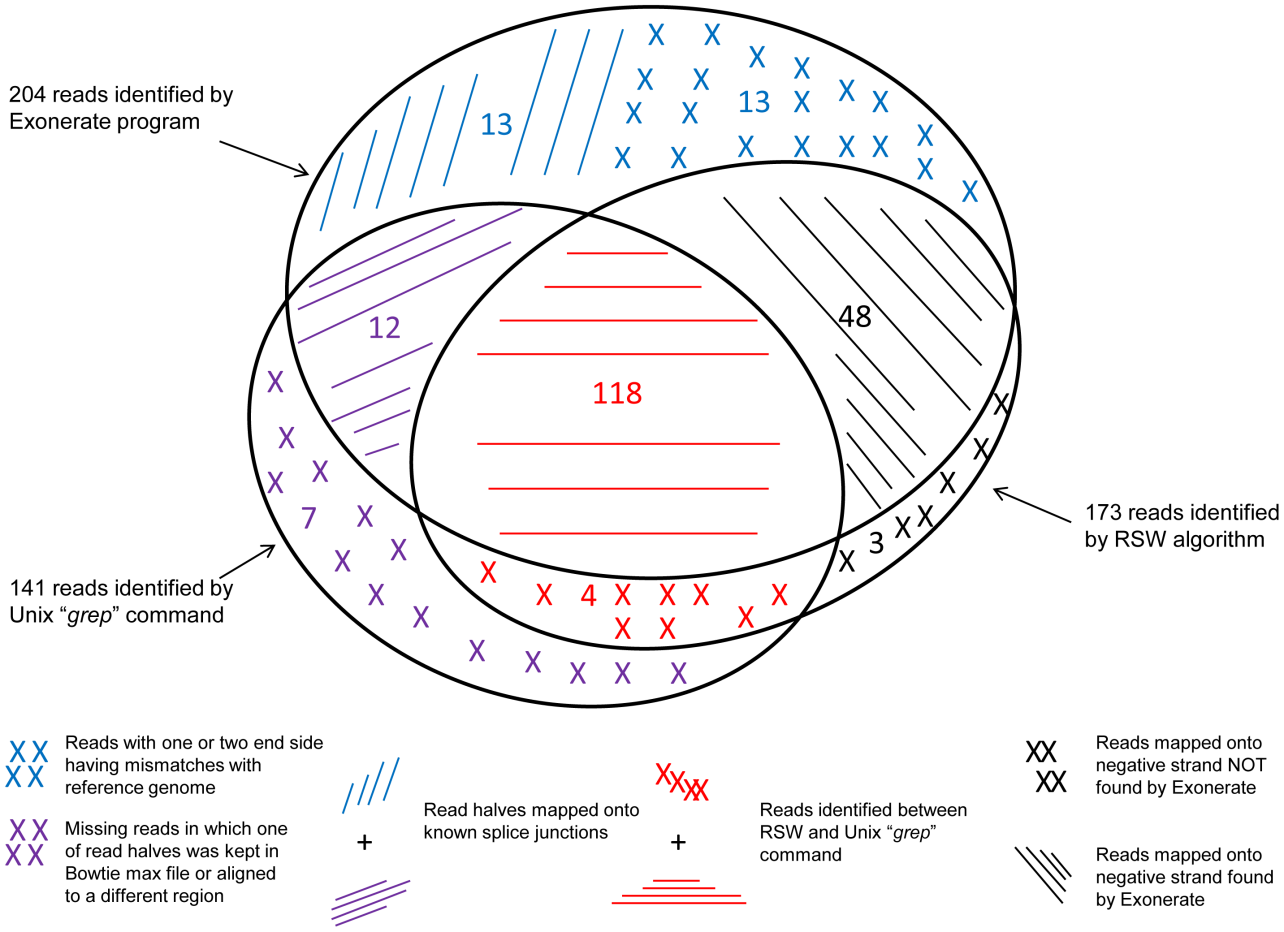
Comparison of supporting reads for *Xbp1*'s 26 nt splice junction site in the Dithiothreitol (Dtt) sample from RSW, Exonerate and the Unix “*grep*” command.

We compared the RSW algorithm results using the Dtt treated Het sample with two different approaches (*grep* and Exonerate) for identifying the number of reads which could be detected spanning the 26 bp spliced region in the positive control gene, *Xbp1*. The Venn diagram comparison of reads detected by the three approaches is shown in Figure 4. We identified 204 and 141 reads spanning the 26 bp spliced region in Dtt-treated Het samples using Exonerate and the Unix *grep* command, respectively, and no reads in either of the KO samples. In contrast, the RSW algorithm identified 173 reads. Among these three methods, 118 unmapped reads are considered in common. Exonerate missed several reads (See Figure 4) that were detected by RSW and vice versa, however, it is important to note that neither Exonerate nor the Unix *grep* approach are applicable for mapping genome-wide data; they can only be used for predefined candidate spliced regions. Conversely, RSW was designed to be utilized on complete unaligned genome-wide data sets.

### Comparison of RSW with BWA

We used the BWA [17] alignment software (backtrack algorithm), which allows for gaps in individual reads, to assess whether the non-canonical spliced region could be detected in this manner. Even with significant modifications to the gap open and extend penalties and the length of gap allowed, we could not identify any of the reads which spanned the 26 nt gap in *Xbp1* in Tg-treated samples. In Dtt-treated samples, BWA only identified 67 of the reads (Table S3). Changing the gap open and gap extension penalty parameters for BWA, we could not identify any more of the reads spanning the splice site. Using *bwa-mem* and *bwa-sw* (alternatives to *bwa backtrack*) did not identify any reads for the 26 bp splice site for either the Dtt or Tg experiments. This is in contrast to RSW, which clearly identified the 26 nt spliced region as the top hit with a significantly higher number of supporting reads in both experiments.

### Comparison of RSW with Bowtie2

Additionally, we used bowtie2 [18] to determine whether the known *Xbp1* 26 nt spliced region could be identified. By using gap extension penalty 1, Bowtie2 recovered most of the reads (166/173 for gap open penalty 15, and 171/173 for open penalty 5) supporting the region of 26 bp in the Dtt sample yet no supporting reads were detected in the Tg data set where RSW reported 21 reads and ranked the *Xbp1* 26 nt region first. Even for the Dtt sample, bowtie2 did not rank the *Xbp1* 26 nt region on top for any parameter combination (at best rank = 62 within chr11 for gap open penalty of 5, with substantially lower rank expected for the entire genome).

### Comparison of RSW with STAR

We applied the STAR (v2.1.1) [19] software with our unmapped read set, which was designed to align non-continuous sequences directly to the reference genome. In both Tg and Dtt samples, STAR did not identify the 26 bp spliced region for *Xbp1* (See Table 3).

**Table 3:** STAR results on the region where *Xbp1* is located.

Sample type	Treatment type	Chromosome						
		First base of the intron						
		Last base of the intron						
		Strand						
		Intron motif						
		Junction type (annotated or unannotated)						
		Number of uniquely mapping reads crossing the junction						
		Number of multi-mapping reads crossing the junction						
		Maximum spliced alignment overhang						
**Tg treated**	**Het**	chr11	5423470 5424241 0	0	1	1	0	3
		chr11	5423471 5424241 1	1	0	7	0	17
		chr11	5424387 5424687 0	0	1	0	1	1
		chr11	5424388 5424687 1	1	0	53	1	17
	**KO**	chr11	5423470 5424241 0	0	1	2	1	3
		chr11	5423471 5424241 1	1	0	15	0	17
		chr11	5424388 5424687 1	1	0	23	0	17
**Dtt treated**	**Het**	chr11	5423470 5424241 0	0	1	18	0	37
		chr11	5423471 5424241 1	1	0	150	0	39
		chr11	5423471 5424310 1	1	0	1	0	13
		chr11	5423471 5430184 1	1	0	1	0	32
		chr11	5424314 5430184 1	1	0	1	0	34
		chr11	5424388 5424687 1	1	0	345	0	39
		chr11	5424388 5424754 1	1	0	2	0	30
		chr11	5424388 5424832 1	1	0	1	0	27
	**KO**	chr11	5423470 5424241 0	0	1	9	0	3
		chr11	5423471 5424241 1	1	0	179	0	39
		chr11	5423471 5424310 1	1	0	4	0	39
		chr11	5424388 5424658 1	1	0	1	0	36
		chr11	5424388 5424687 1	1	0	319	0	39
		chr11	5424388 5424754 1	1	0	4	0	37
		chr11	5424388 5424832 1	1	0	1	0	27
		chr11	5424388 5425015 1	1	0	1	0	23

### Running RSW on SKBR3 human Chromosome 17 breast cancer paired-end RNA-Seq data set

The SKBR3 proteomic analyses of chromosome 17 reported 217 distinct protein isoforms from 108 genes with transcript and peptide evidence of novel, alternative splicing; these 108 genes are annotated in the Ensembl database as genes with more than one protein-coding transcript [23]. All of the 108 genes were identified as having novel spliced regions by RSW (Table 4). Among the 217 Ensembl protein isoform IDs reported by the SKBR3 proteomics analysis, 214 (98.6%) of these isoforms were found to share at least one common exon junction with our RSW results (Table S4). RSW results also indicated that these 108 genes expressed in the SKBR3 cell line have a large number of novel spliced regions when compared to other genes (data not shown). A total of 261 novel splice regions from this 108 gene data set were supported by two or more reads. A list of novel candidate spliced regions is provided in Table S5.

**Table 4:** Counts of spliced regions detected by RSW and proteomics from the SKBR3 cell line.

dataset	Read 1 only		Read 2 only		Both Read1&2
Gene Set	107	582	108	620	656
	Both methods	RSW only	Both methods	RSW only	RSW only
Novel Isoform	398	1022	435	1105	**424**
Known Isoform	868	2634	956	3145	**2692**
Total Isoform	1266	3656	1391	4250	**3116**

Read 1 refers to the first half of each paired-end read; Read 2 refers to the second half of each paired-end read. Notes: 107 Gene Set (Found by both proteomics and RSW methods using the first half of each paired-end read); 582 Gene Set (Found by RSW only using the first half of each paired-end read); 108 Gene Set (Found by both proteomics and RSW methods using the second half of each paired-end read); 620 Gene Set (Found by RSW only using the second half of each paired-end read); Both Read1&2 (Common junctions identified by both halves of paired-end data. They are located within the same gene, have the same splice length, and share the same splice boundary).

## Discussion

### Technical considerations for the RSW pipeline

Using Bowtie alignment, the unmapped reads dataset changes when different alignment parameters, e.g., –m, -k, and/or –v, are used. In our case, after testing several different parameter combinations, it appears that the default settings work well for the data. However, better combinations may exist, and different parameter combinations may be optimal for different species or datasets. In our pipeline, we fixed certain parameters, such as the minimum split read size after exploring a limited set of reasonable values. It is worth considering how these choices affect the outcome for different datasets. For example, there is a trade-off between the lower/upper bound split size *vs* alignment sensitivity. By allowing a read half to map only up to 10 locations, we are specifying that the chance that it maps near the other read split half and in the correct configuration is small; if the minimum read split size is increased, then fewer read halves will map to >10 locations. Similarly, there is a trade-off between shorter/longer consolidation slip/buffer size. We used 5 bp because the minimum read half length used was 11 bp for both runs and therefore our buffer size is half of the minimum read half length. More or fewer supporting reads may be reported if different cut-off criteria are applied; these can be adjusted to achieve the desired balance between sensitivity and specificity in the specific application. Several factors will also influence the detection power of RSW. These factors include: total read coverage (total number of experimental reads); strength of the splicing event(s), i.e. the proportion of the target transcripts that are spliced, with spicing events that occur at a higher rate being easier to detect; gene expression level, with splicing of more highly expressed genes being easier to detect; and stringency of filtering parameters used.

### Maximum distance between two split halves

For mouse MEF data, we selected a 40 kb distance between two split read halves to serve as the maximum expected splice length of an IRE1α target. The distance of 50 kb was used for human breast cancer data. However, the question remains as to whether this was the best selection criterion for read pairs. We chose 40 kb for mouse (50 kb for human) as an arbitrary maximum because the average gene length is 47 kb for mouse genome (56 kb for human genome) based on the UCSC mm9 refFlat file in mouse and we assumed the maximum splicing length for a given gene would be less than the average total gene length. Using a shorter maximum distance will minimize false positives, while a longer maximum distance may increase sensitivity. If the maximum split length is increased, users may compensate by increasing the minimum split half size and/or decreasing the maximum number of mapping places in order to avoid an excess of false positive mappings in that region. We also used a very wide range (2 bp, 40 kb) of possible splice lengths, since we had data for two *Ire1α* null cell lines that we could intersect to reduce false positives. Users who do not have this advantage my well want to reduce the range of acceptable splice lengths. Although only recommended for longer reads under stringent parameter conditions, it is also possible to run RSW a second time without any filter on split length to interrogate whether trans-mRNA splicing may occur.

### The comparative performance of the RSW algorithm

The splitting of reads into multiple read half pairs substantially increased the dataset size. Assuming all of the 218 unmapped reads supporting the known *Xbp1* 26 nt splice site are true positives, RSW has an estimated 79% (173/218 overlap between RSW and both *grep* and Exonerate) true positive rate (sensitivity). The false positive rate of RSW for the *Xbp1* site is estimated to be 0%, since all reads identified by RSW appear to be true positives at least with regard to their sequencing. When compared to the results generated from the Unix *grep* command, RSW only missed reads in which one of the read halves was kept in Bowtie's max file (The output file contains all reads with a number of valid alignments exceeding the limit set with the –m option) or aligned to a different region (7 reads). Exonerate [20] results included reads where one or both read ends from a pair mapped onto a nearby known splice junction (25 reads), and thus were not part of the unmapped reads that RSW was starting from. The remaining 13 unmapped reads identified using Exonerate are possible false positives because those reads have one or two mismatches beyond the 33 bp query sequences of the Exonerate input.

Given the reasons above, we conclude that, by using different parameter settings for Bowtie [24] aligner along with RSW, we achieved better sensitivity than the other three methods for detection of *Xbp1*'s IRE1α dependent intron. BWA [17] was not able to detect the relatively large 26 nt splice as a gap in any of the reads. It is worth stressing that both Exonerate and use of the Unix *grep* command require prior knowledge of the flanking sequence to check against unmapped reads, whereas our RSW uses the unmapped read set to check against the entire reference genome. At best, Bowtie2 was only able to detect the 26 nt splice region in the Dtt experiment, and even then it was ranked low (62 just within chr11). Thus, the RSW method performs best for genome wide *de novo* discovery of such short, non-canonical, or otherwise previously unknown, spliced regions. Our pipeline also compares and selects both samples' information side-by-side. A similar analysis using our RSW algorithm can be extended to other species, as demonstrated with our analysis of the SKBR3 human breast cancer cell line.

## Conclusions

The positive control for our application, the *Xbp1* 26 nt non-canonical spliced region, was clearly detected in *Ire1*α heterozygous samples from treated samples but not in the negative control samples, and was our top hit for an *Ire1*α target splice site with both ER stress-inducing treatments. We identified a small number of other potential novel splice locations, each with variable size. However either the number of reads supporting these splices was much lower than observed for *Xbp1*, the splice length was likely too small or large to be a true positive, the results were only found in one treatment or the gap was genomic in origin. We have also demonstrated the application of RSW on well-characterized splicing events (via mass spectrometry) in the SKBR3 human cancer cell line. Using this method to detect non-canonical or otherwise unknown spliced regions in unmapped mRNA-Seq data, we conclude that *Xbp1* mRNA is likely the only significant splicing target of IRE1α in the MEF transcriptome. However, it is possible that other IRE1α-dependent splice targets escaped detection if they form after the peak of *Xbp1* splicing. Therefore, future experiments using longer ER stress treatments of control and *Ire1α* knock-out human secretory cells are planned.

## Supporting Information

Table S1 **Reads mapped by the RSW pipeline on Xbp1's 26 nt spliced region in the Tg and Dtt** ***Ire1α^+/−^*** **samples.**
(XLSX)

Table S2 **A list of genes with novel spliced regions detected by RSW and their read supporting information.**
(XLSX)

Table S3 **A list of reads supporting** ***Xbp1*** **26 bp spliced region identified by BWA.**
(XLSX)

Table S4 **Overlap between RSW results with exon junctions of protein isoform IDs identified by another SKBR3 study.**
(XLSX)

Table S5 **A list of novel splice junctions reported by RSW algorithm for human breast cancer chromosome 17 RNA-Seq data.**
(TXT)
